# Effects of *Lacticaseibacillus paracasei* K56 on perceived stress among pregraduate students: a double-blind, randomized, placebo-controlled trial

**DOI:** 10.3389/fnut.2025.1544713

**Published:** 2025-03-12

**Authors:** Yiran Guan, Ruixin Zhu, Wen Zhao, Langrun Wang, Li You, Zhaozhong Zeng, Qiuyue Jiang, Zeyang Zhu, Jiayu Gou, Qi Zhang, Jie Guo, Keji Li, Liang Zhao, Yixuan Li, Pengjie Wang, Bing Fang, Weilian Hung, Jian He, Liwei Zhang, Ran Wang, Jingjing He

**Affiliations:** ^1^Key Laboratory of Functional Dairy, Co-Constructed by Ministry of Education and Beijing Government, Department of Nutrition and Health, China Agricultural University, Beijing, China; ^2^College of Physical Education and Health, Chongqing College of International Business and Economics, Chongqing, China; ^3^National Center of Technology Innovation for Dairy, Hohhot, China; ^4^Department of Nutrition and Food Hygiene, School of Public Health, Peking University, Beijing, China; ^5^Beijing Advanced Innovation Center for Food Nutrition and Human Health, China Agricultural University, Beijing, China; ^6^Research Center for Probiotics, China Agricultural University, Beijing, China

**Keywords:** *Lacticaseibacillus paracasei* K56, pregraduate students, pressure, gut microbiota, butyric acid

## Abstract

**Background:**

Globally, master’s and doctoral students, especially pregraduate students, are under great pressure. Probiotics are emerging as a promising intervention to improve mental health via gut-brain axis.

**Objective:**

The aim of this study was to explore the impact of *Lacticaseibacillus paracasei* K56 supplementation on perceived stress among pregraduate students.

**Methods:**

We conducted a double-blind, randomized, placebo-controlled trial in 120 healthy master’s and doctoral students who faced graduation. Participants were randomly assigned to either probiotics (containing *Lacticaseibacillus paracasei* K56 6 × 10^10^ CFU / d) or placebo group for 2 weeks intervention. The main outcome was perceived stress assessed using Cohen’s Perceived Stress Scale-10 (PSS-10). The secondary outcomes were stress, depression, and anxiety assessed by Depression, Anxiety and Stress Scales (DASS), gastrointestinal symptoms, and sleep evaluated by corresponding scales. These outcomes were assessed at baseline, 1, and 2 weeks. Pre- and post-treatment serum biomarkers, gut microbiota composition and metabolites were also detected.

**Results:**

There was no difference in changes of PSS-10 scores from baseline to 2 weeks between the K56 groups and the placebo [mean (standard error): −1.68 (0.48) vs. -0.39 (0.46), *p* = 0.055]. Furthermore, the K56 group exhibited superior reductions in both stress [−2.15 (0.38) vs. -0.96 (0.49), *p* = 0.035] and anxiety symptoms [−1.54 (0.32) vs. 0.53 (0.43), *p* = 0.003] via DASS compared with the placebo group. Additionally, those receiving K56 also experienced improved sleep quality (*p* = 0.010) and elevated levels of serotonin (5-HT) (*p* = 0.038) compare to placebo group. Moreover, taking probiotics K56 could modulate the pressure-induced changes in gut microbiota composition, particularly by increasing the beneficial bacteria (*Lacticaseibacillus* and *Lacticaseibacillus paracasei*), while suppressing suspected pathogenic bacteria (*Shieglla* and *Escherichia_coli*). Metabolomic analysis revealed an increased in metabolites, especially butyric acid in the K56 group (*p* = 0.035). Notably, there was a significant negative correlation between relative abundance of *lactobacillus* and stress-related symptoms, whereas butyric acid showed a significant positive correlation with *lactobacillus* abundance level.

**Conclusion:**

This study suggested the potential benefits of K56 supplementation in alleviating stress and significant effect in reducing anxiety and insomnia among master’s and doctoral students, which may be attributed to K56-induced changes in microbial composition and butanoate metabolism.

**Clinical trial registration:**

Chictr.org.cn, identifier ChiCTR2300078447.

## 1 Introduction

Stress is a universal experience. Short-term stress can be beneficial, serving as a motivator and enhancing resilience (1), while chronic stress has detrimental effects on both physical and mental well-being. The brain is particularly susceptible to the adverse impacts of chronic stress, increasing vulnerability to neuropsychiatric disorders such as anxiety and depression (2). The mental health problems of master’s and doctoral students have been getting worse in recent years (3, 4). In a meta-analysis of 32 studies, it was found that approximately 17% of doctoral students experienced anxiety, while around 24% had depression (5). The latest survey of 6,300 graduate students worldwide revealed that 36% doctoral students had sought help for anxiety or depression related to academic difficulties (6), with thesis writing and defense being the major sources of stress (7). Effective approaches to relieve the psychological stress of master’s and doctoral students are therefore urgently needed.

Gut microbiota and the gut-brain axis play a vital role in mental health and cognitive function. Signals from the gut, such as neural pathways, cytokines, hormones and neuropeptides, can influence emotional behavior and stress response systems (8). Probiotics are live microorganisms that have the ability to improve gut, brain and mental health by manipulating gut microbiota and regulating the gut-brain axis (9). Specifically, *Bifidobacterium lactis* CNCM I-2494 has been demonstrated to reduce stress-induced glucocorticoid and inflammatory cytokine responses, thereby alleviating depression and anxiety-related behaviors (10). Additionally, *Lactobacillus helveticus* NS8 has shown improvement in chronic stress-induced behaviors such as anxiety and depression (11). In recent clinical studies, *Lacticaseibacillus paracasei* Lpc-37® was shown to reduce perceived stress in healthy adults (12), but had no effect on stress, mood, or anxiety among healthy college students experiencing chronic academic stress (13). Given the conflicting findings on probiotics’ impact on mental health in humans and considering the significant mental burden faced by master’s and doctoral students, further clinical trials are warranted to assess the effects of probiotics on mental health in this specific population.

*Lacticaseibacillus paracasei* K56, extracted from the gastrointestinal tract of healthy infants in China, is a novel strain of probiotics that has been deemed safe for human consumption (14). Animal studies have demonstrated that K56 exerts regulatory effects on the metabolism of gut microbiota and short-chain fatty acids (15). In addition, co-administration of K56 with coix seed has shown promising results in ameliorating chronic inflammation in obese mice (16). Clinical trials have also shown that supplementation with *Lacticaseibacillus paracasei* K56 modulated gut microbial diversity and composition in adults with obesity (17). It is hypothesized that K56 may relieve stress through modulation of the gut-brain axis by improving gut microbiota-mediated inflammatory immune responses. Therefore, this study aimed to investigate the effects and underlying mechanisms of K56 on chronic stress and other mental health outcomes among master’s and doctoral students.

## 2 Materials and methods

### 2.1 Study design

This was a randomized, double-blind, placebo-controlled clinical trial with two parallel arms (allocation ratio 1:1). Prior to the recruitment, the study protocol and the informed consent form were reviewed and approved by the Human Research Ethics Committee of China Agricultural University on November 15, 2023 (CAUHR-20231202) and was registered in the Chinese Clinical Trial Registry (ChiCTR2300078447). The study was conducted in Beijing, China in accordance with the Declaration of Helsinki (18), following all applicable laws and regulations for clinical research in China.

**Figure 1:**
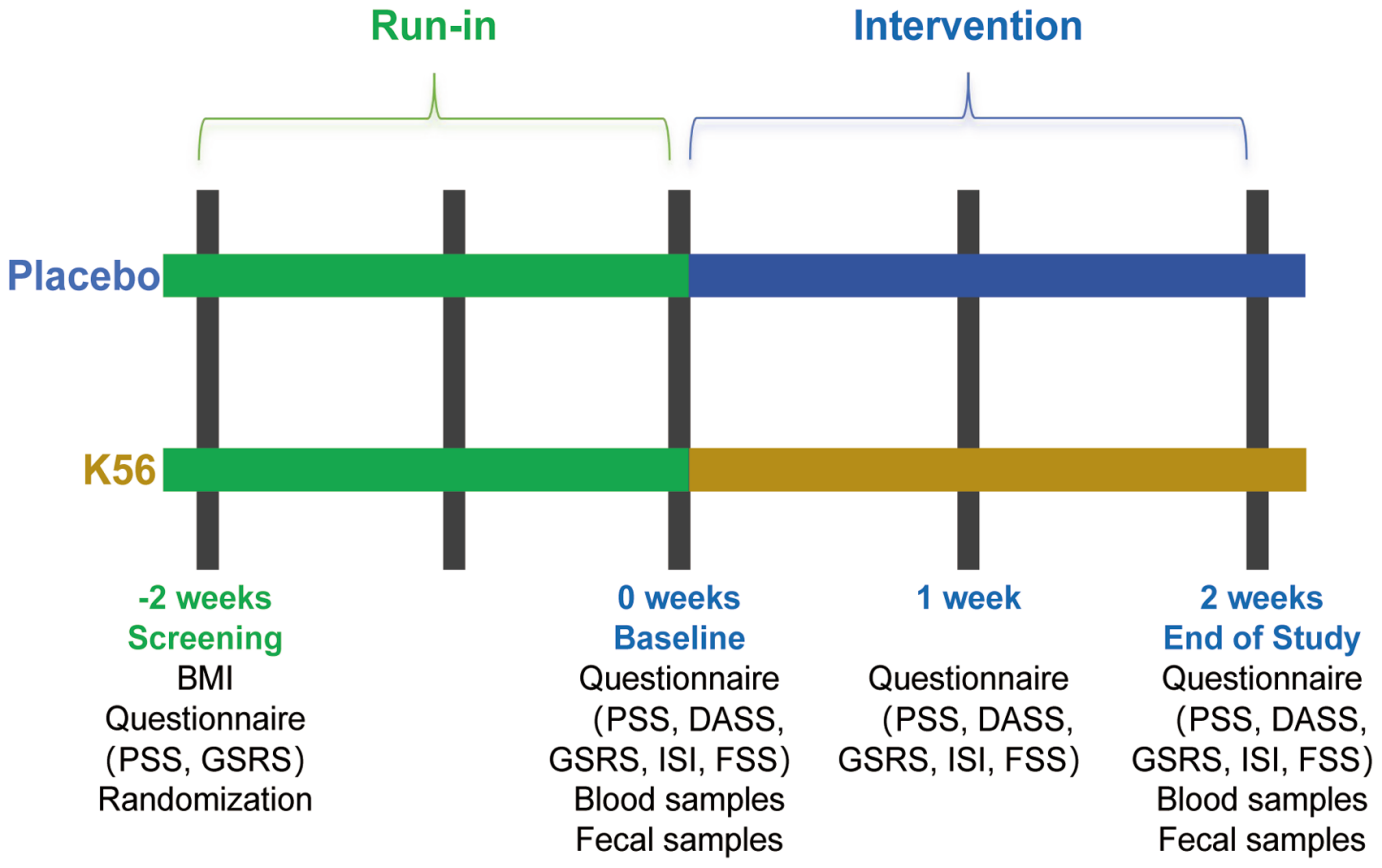
Study design. BMI, Body mass index; PSS, Cohen’s perceived stress scale; DASS, the depression, anxiety and stress scales; GSRS, the gastrointestinal symptom rating scale; ISI, the insomnia severity index; FSS, the fatigue severity scale.

The study design included a two-week run-in period prior to the intervention during which randomized participants were not permitted to consume products containing concentrated sources of probiotics and/or prebiotics (Figure 1). This was followed by a two-week intervention phase involving the administration of either test products containing K56 or the placebo products between weeks 0 and 2. Randomized participants were provided with fecal collection kits and were instructed to collect their fecal sample as close to the scheduled study visit as possible before the weeks 0, 1, and 2. Additionally, participants were required to complete weekly online questionnaires assessing symptoms related to stress, anxiety, depression, sleep quality, fatigue levels, and gastrointestinal issues from weeks 0 to 2. Prior to the formal collection of stool samples and completion of questionnaires, all participants underwent standardized training conducted by the researchers.

### 2.2 Study participants and randomization

Participants were recruited from universities and academic institutions via targeted advertising. A total of 120 eligible participants provided informed consent forms and were randomized into 2 intervention groups. Inclusion criteria included men or women, aged 18–35 years old, master’s or doctoral students who were expected to graduate between June 2024 and July 2024, and have a moderate stress level (14–26 scores) based on Cohen’s Perceived Stress Scale (PSS-10) (19). Exclusion criteria include individuals who: (1) had other stress sources apart from graduation, such as family, illness, finance, etc. (2) had mental disorders (e.g., depression, anxiety disorder, bipolar spectrum disorder, or schizophrenia), severe gastrointestinal diseases (e.g., gastric ulcers, Crohn’s disease, or ulcerative colitis) or chronic illness (e.g., diabetes, liver disease, kidney disease, or heart disease); (3) were on psychoactive medication, gastrointestinal medication, dietary supplements, or probiotics; (4) had a recent history of antibiotic therapy. The full description of eligibility criteria is provided in Supplementary Appendix. Randomization was performed upon checking of the inclusion and exclusion criteria. Once the participants were enrolled by staff, the data statisticians employed a dynamic randomization method to allocate eligible individuals in a 1:1 ratio to either the probiotics group, which received *Lacticaseibacillus paracasei* K56, or the placebo group. This allocation was stratified based on gender, academic degree, baseline Body Mass Index (BMI), and baseline Cohen’s Perceived Stress Scale (PSS) score, ensuring a balanced distribution across key variables. Both the researchers and participants remained blissfully unaware of the group assignments, maintaining the integrity of the blind study design.

### 2.3 Study products

During the two-week intake period, participants in the K56 group consumed 1 bottle of fermented milk beverage per day containing *L. paracasei* K56 (6 × 10^10^ CFU live cells per 100 mL, deposit No. CGMCC 15139, provided by Inner Mongolia Yili Industrial Group Company, China). The placebo products were dairy products without K56. The placebo products had the same packaging, color, texture, taste, and nutritional content as the test counterparts. Both the test and placebo products contained the following additives authorized for use in food for human consumption: skim milk powder, water, edible glucose, white sugar, lactic acid or food flavor (see Supplementary Appendix). A two-week supply was provided to participants at 0 weeks. Participants were asked to store the dairy products at 0 to 10°C. Participants were instructed to consume 100 mL of study products everyday at dinner time.

The identity of the study products was blinded to participants, site staff, the principal investigator, data manager, and statistician in the trial.

### 2.4 Study outcomes

#### 2.4.1 Primary outcome

The primary outcome was changes in perceived stress levels from baseline to week 2 in the test products versus the control products (placebo). The perceived stress levels were assessed using the Perceived Stress Scale-10 (PSS-10) questionnaire and participants with moderate levels of stress were recruited to join the study. PSS-10 consisted of 10 items rated on a 5-point Likert scale. Among these items, six were negatively stated (0 = never, 1 = almost never, 2 = sometimes, 3 = fairly often, 4 = very often) while the remaining four were positively stated (items 4, 5, 7, and 8) and reverse-scored (0 = very often, 1 = fairy often, 2 = sometimes, 3 = almost never, 4 = never). The total score was calculated by summing up responses to all ten items, with scores ranging from 0–13, 14–26, and 27–40 indicating low, moderate, and high levels of perceived stress, respectively. Chinese translations and verified versions of the PSS-10 questionnaire were used to assess participants’ perception of stress (20). The Chinese version of the scale demonstrated good reliability (Cronbach’s *α* = 0.86, test–retest = 0.68) and validity (factor loadings >0.5).

#### 2.4.2 Secondary outcome

The secondary outcomes assessed in this study included changes in perceived stress levels (measured by PSS scale scores) from baseline to week 1, as well as changes in various stress-induced symptoms including anxiety, depression, sleep quality, fatigue levels, and gastrointestinal issues between the test product group versus the control product (placebo) group. Additionally, alterations in serum biomarkers, gut microbiota composition, and metabolite profiles were evaluated from baseline to weeks 1 and 2 between groups.

#### 2.4.3 Self-reported stress-induced symptoms

The Depression, Anxiety and Stress Scales – 21 items (DASS-21) was used to evaluate negative emotional states of depression, anxiety, and stress (21) during the past week. The DASS-21 is a shorter version of the 42-item DASS, and has been shown to have good reliability and validity properties in clinical populations (22). The revised Chinese version of the Depression Anxiety and Stress Scale (DASS-21) (23) was used in this study. This scale included 21 items with 7 items each for depression, anxiety, and stress. The cut-off values for depression were as follows: below 10, mild depression; 11–14, moderate depression, and 15–21, severe depression. The cut-off values for anxiety were as follows: 0–8, mild anxiety; 9–10, moderate anxiety; and 11–15, severe anxiety. The cut-off values for stress were as follows: 0–15, mild pressure; 16–19, moderate; and 20–26, severe pressure.

The Gastrointestinal Symptom Rating Scale (GSRS) is a self-report, 15-item questionnaire that measures the severity of a wide range of gastrointestinal symptoms during the past week (24). Items are rated on a 7-point scale ranging from no discomfort at all [1] to very severe discomfort [7]. A total score is calculated by summing the scores of all items. In addition to a GSRS total score, 4 symptom clusters can also be calculated comprising scores for “Bowel dysfunction syndrome,” “Indigestion syndrome,” “Dyspeptic syndrome,” and “Abdominal pain syndrome.”

The Insomnia Severity Index (ISI) (25) is a 7-item scale that was designed to assess the severity of both nighttime and daytime components of insomnia during 2 past weeks. The sum score of the ISI ranges from 0 to 28 and higher scores indicate worse insomnia. Chinese translations and verified versions of the ISI questionnaire were used in this study to assess the sleep quality, which has adequate psychometric properties and is sensitive to treatment response (Cronbach’s *α* = 0.83, test–retest = 0.79, factor loadings >0.5) (26).

The Fatigue Severity Scale (FSS), a Likert scale consisting of 9 items, was used to assess fatigue severity and functionality during the past week (27). Items were rated on a scale of 1 to 7 according to their level of agreement with a given statement and included statements such as “Fatigue brings frequent discomfort” or “Fatigue affects my physical ability.” The FSS score is the mean score of the nine items, and a higher FSS score indicates more fatigue. An FSS score of 3 or 4 has previously been used as cut-off for fatigue, and ≥ 4 was used in this study as a conservative approach (28).

All the questionnaires were used for assessment at baseline (week 0), one week after intervention (week 1), and at the end of intervention (week 2).

##### 2.4.3.1 Serum biomarkers

All participants were invited to provide blood samples voluntarily. Blood samples (5 mL) were drawn from an antecubital vein directly before the participants had breakfast, 3 times throughout the study (0, 1 and 2 weeks). Serum samples were analyzed for the concentrations of stress hormone cortisol, serotonin, interleukin-1β and interferon (IFN)-*γ* using enzyme-linked immunoabsorbent assay (ELISA) kits (Shanghai Yuanju Biotechnology, China) following the manufacturer’s instructions.

##### 2.4.3.2 Fecal sample metagenome and metabolome detection

Before the intervention, 30 randomly selected participants in each group were instructed to collect their fecal samples, who also provided stool samples after the intervention, resulting a total of 120 fecal samples. Fresh fecal samples were collected in the morning prior to any food consumption and placed in sterile retention bottles. Subsequently, the stool samples were immediately placed on ice, transported to the laboratory within 1 h, and frozen at −80°C for subsequent use (29). Importantly, fecal samples were homogenized by Bertin Precellys Evolution sample homogenizer (Bertin Technologies SAS, France) (30, 31), and then the homogenized fecal samples were randomly weighed for further index detection. The fecal DNA extractions were processed following the MetaHIT protocol, then Single-end metagenomics sequencing were performed using BGISEQ-500 platform. And fecal metabolite features were analyzed using a UHPLC system (Vanquish, Thermo Fisher Scientific) with a UPLC BEH Amide column (2.1 mm × 100 mm, 1.7 μm) coupled to the Q Exactive HFX mass spectrometer (Orbitrap MS, Thermo) (32). More detailed methods were shown in Supplementary Methods.

### 2.5 Adverse events

Adverse events (AE) were assessed in each intervention group at each visit with open, standardized questions such as “Have you had any health problems since your last visit?” Additionally, participants were asked to record any occurring AE as follows: description of the event, onset (date and time), severity, treatment and outcome, causal relationship with the study product, whether to withdraw the experiment accordingly. The PI classified causality (definitely, probably, possibly, unlikely, not related, not assessable) and whether it constituted a serious adverse event (SAE) or not. Any AEs still ongoing at study completion on 2 weeks were followed up to 14 days after 2 weeks.

### 2.6 Sample size calculation and statistical analysis

The sample size was estimated based on the PSS score. According to the results from a previous study (12), the standard deviation corresponding to a 2.4-point reduction in stress after taking *Lactobacillus paracei* for 5 weeks is approximately 7.4. We used the Repeated Measures module in PASS software with 3 repeated measurements (33). Based on sample size/power analytic methods for repeated measures analysis (34, 35), using a two-sided test with a Type I error of 0.05 and an 80% power, with the covariance type set to simple, it was calculated that at least 50 subjects per group and 100 in total were required. Considering a 20% dropout rate, at least 60 subjects per group and 120 in total needed to be recruited.

Data were analyzed using SPSS version 28.0 (SPSS Institute, Chicago, IL, USA) and figures were created with GraphPad Prism 8 (GraphPad Software, The North Parker, USA). All tests were two-sided with *p* < 0.05 as considered statistically significant. In descriptive statistical analysis, continuous variables with normal or approximately normal distribution are described by mean (standard deviation), and continuous variables with skewed distribution are described as medians and interquartile ranges. The effect sizes for the differences before and after the intervention within the group or between the groups were expressed as mean values accompanied by their standard errors (SE). For the primary outcome and secondary outcomes, independent t test or Wilcoxon rank sum tests were conducted to compare changes between the K56 group and the placebo group at 1 and 2 weeks from baseline. All secondary outcomes were considered exploratory. Pre- and post-treatment changes within each group were analyzed using paired t tests or Wilcoxon rank sum tests. In addition to the analysis in the general population, to further explore the potential heterogeneity of intervention effects, subgroup analyses were conducted on symptom outcomes two weeks post-intervention, stratified by sex (female and male) and age group (younger: ≤24 years; older: >24 years, with the median age as the cutoff).

For metagenomic analyses, Statistical analyses were mainly performed in the program R version 3.4.3. Alpha diversity was calculated as the Shannon index (36), while Bray–Curtis dissimilarity (37) was used to compute the beta diversity. Splinectome R was used for the longitudinal microbiome group comparison (38). The metabolomic data were subjected to multivariate analysis using SIMCA 16.0.2 software package (Sartorius Stedim Data Analytics AB, Umea, Sweden). An unsupervised principal component analysis (PCA) and a supervised model of orthogonal projections to latent structures-discriminate analysis (OPLS-DA) were performed. To further elucidate the potential role of microbiota and metabolites, Spearman correlation analyses were performed to investigate the associations between phenotypic characteristics, microbiota and metabolite profiles.

## 3 Results

### 3.1 Participants’ baseline characteristics

**Figure 2:**
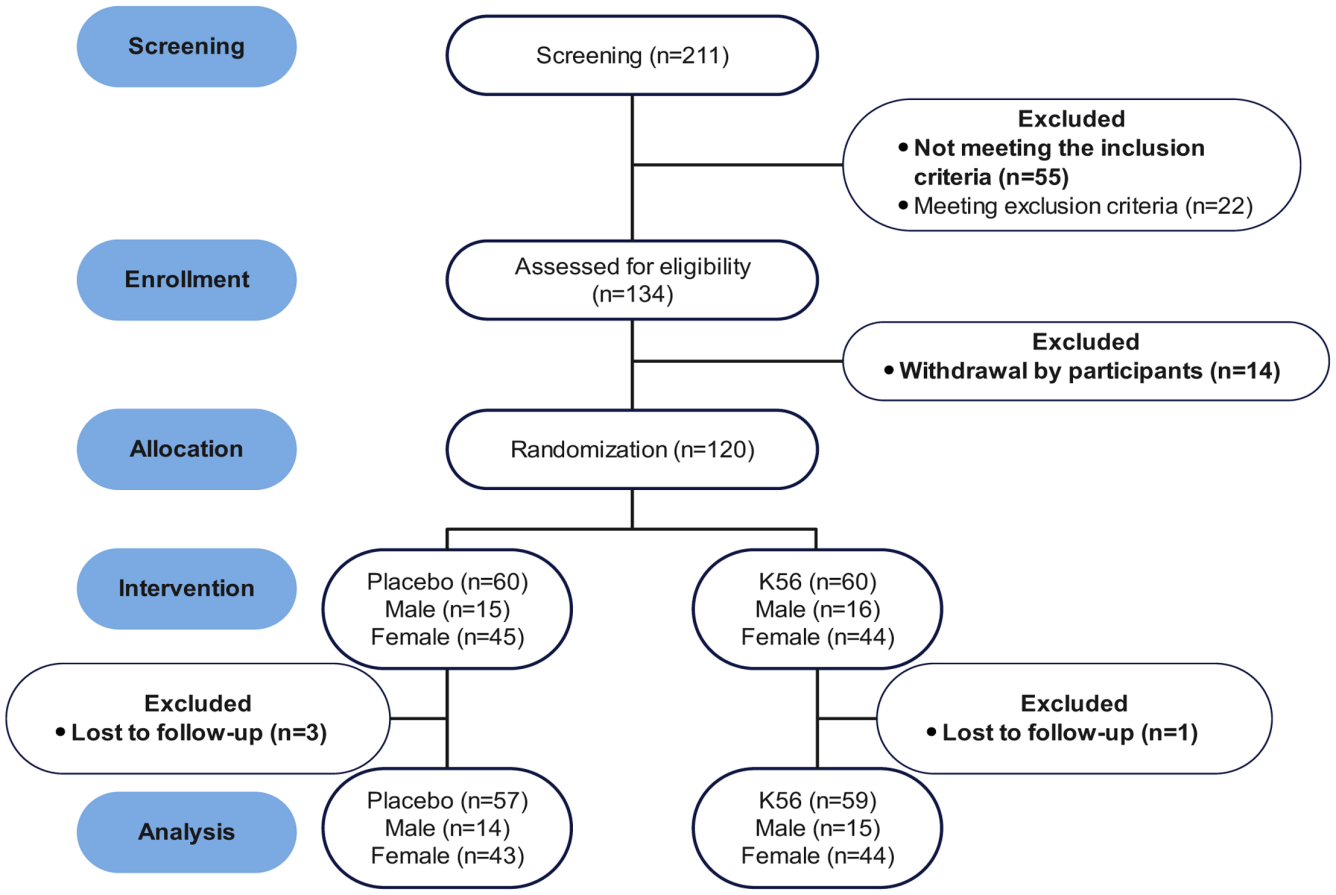
CONSORT flow diagram.

A total of 211 participants showed interests and 120 of them were eligible to enter the trial, with 60 participants in each intervention group. Four participants were lost to follow-up during the intervention, resulting in a final cohort of 116 participants who completed the study and were subsequently incorporated into the analytical dataset. Figure 2 shows the CONSORT flow chart. There were no statistically significant differences in age, sex ratio, BMI, PSS, DASS, GSRS, ISI, or FSS global scores between the two groups at baseline (Table 1). The study began on December 24, 2023 and completed on January 21, 2024.

**Table 1:** Participants’ baseline characteristics (*n* = 120).

	Placebo	K56	*P* Value
Sample size (n)	60	60	
Sex (male/female)	15/45	16/44	0.835
Age (years)	24.4 ± 2.3	24.4 ± 2.2	0.968
Height (cm)	165.8 ± 7.1	167.1 ± 7.5	0.344
Weight (kg)	61.4 ± 16.1	60.9 ± 11.3	0.854
BMI (kg/m^2^)	22.2 ± 4.9	21.8 ± 3.6	0.586
PSS-10 score	20.5 ± 3.4	20.0 ± 3.6	0.464
DASS 21 score			
Depression	5.0 (3.0, 7.0)	6.0 (3.0, 8.0)	0.611
Anxiety	5.0 (3.0, 7.0)	5.0 (3.0, 7.0)	0.373
Stress	8.0 (5.0, 10.0)	7.0 (6.0, 10.0)	0.802
GSRS score	33.6 ± 10.3	33.2 ± 9.9	0.814
Abdominal pain	2.0 (1.0, 3.0)	1.0 (1.0, 2.0)	0.290
Indigestion	9.0 (7.0, 10.0)	8.0 (6.0, 10.0)	0.532
Dyspeptic	10.0 (8.0, 13.0)	9.0 (8.0, 12.0)	0.964
Bowel dysfunction	12.0 (9.0, 16.0)	12.0 (9.0, 15.0)	0.854
ISI score	7.2 ± 4.3	8.3 ± 4.7	0.213
FSS score	44.5 ± 10.0	43.5 ± 10.4	0.604

### 3.2 Primary outcome

**Figure 3:**
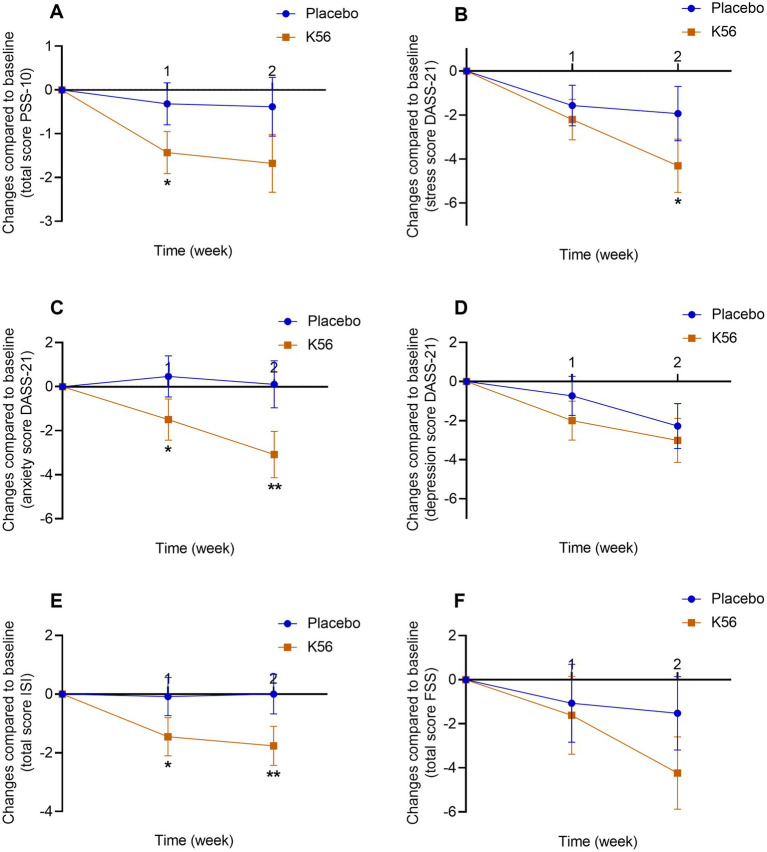
Changes and standard errors in self-report psychological symptoms between the K56 group and the placebo group from baseline over 2 weeks. **(A)** PSS-10 score, **(B)** DASS-21 stress score, **(C)** DASS-21 anxiety score, **(D)** DASS-21 depression score, **(E)** ISI score. **(F)** FSS questionnaire score. The asterisk denotes the statistical significance of the differences between groups in changes observed at 1 week and 2 weeks compared to the baseline. *p < 0.05, **p < 0.01. PSS, Cohen’s perceived stress scale; DASS, the depression, anxiety and stress scales; GSRS, the gastrointestinal symptom rating scale; ISI, the insomnia severity index; FSS, the fatigue severity scale.

Changes in PSS-10 scores from baseline between the 2 intervention groups are shown in Figure 3A. At the end of treatment, a greater reduction in scores was observed in the K56 group; however, no significant difference was found between the two groups [mean (SE): −1.68 (0.48) vs. -0.39 (0.46), *p* = 0.055, Figure 3A].

The sex-specific subgroup analysis revealed that the 2-week K56 intervention led to a significant reduction in PSS scores compared to the placebo group among females [−1.75 (0.55) vs. 0.09 (0.50), *p* = 0.016, Supplementary Table S4], whereas no significant intergroup differences were observed in males. Furthermore, age-based subgroup analysis found no significant effect of K56 on PSS scores in either the younger or older participants.

### 3.3 Secondary and ancillary outcomes

At 1 week, the K56 group had a greater reduction in PSS-10 score compared with the placebo group [−1.43 (0.36) vs. -0.32 (0.32), *p* = 0.022, Figure 3A]. Although both treatments significantly reduced DASS-21 stress scores, stress dropped more sharply in the K56 group compared with the placebo group at 2 weeks [−2.15 (0.38) vs. -0.96 (0.49), *p* = 0.035, Figure 3B]. The K56 group had a greater decrease in DASS-21 anxiety scores from baseline at 1 week [−0.75 (0.35) vs. -0.23 (0.32), *p* = 0.038, Figure 3C] and 2 weeks [−1.54 (0.32) vs. 0.53 (0.43), *p* = 0.003, Figure 3C] compared with the placebo group. There were no significant differences in DASS-21 depression score from baseline between the K56 group and the placebo group over 2 weeks [−1.00 (0.38) vs. -0.37 (0.33), *p* = 0.207 for 1 week, −1.51 (0.37) vs. -1.14 (0.43), *p* = 0.518 for 2 weeks, Figure 3D]. The results of the subgroup analysis indicated that the effect of 2-week K56 intervention on depression scores of the DASS scale were not statistically significant across any subgroups (males, females, young adults, and older adults), aligning with the findings in the general population. In contrast, for DASS stress and anxiety scores, the K56 effect (vs placebo) was more pronounced in females[stress: −2.22 (0.39) vs. -0.65 (0.44), *p* = 0.009; anxiety: −1.70 (0.39) vs. 0.21 (0.39), *p* = 0.001; Supplementary Table S4] and younger participants [stress: −2.49 (0.47) vs. -0.52 (0.57), *p* = 0.009; anxiety: −1.78 (0.37) vs. 0.32 (0.52), *p* = 0.001; Supplementary Table S5], whereas it was not observed in males and older participants.

As shown in Figure 3E, a greater reduction in ISI score was observed in the K56 group than the placebo group at both visits [−1.45 (0.47) vs. -0.08 (0.45), *p* = 0.038 for 1 week, −1.76 (0.48) vs. 0.00 (0.48), *p* = 0.010 for 2 weeks]. The placebo group maintained subclinical insomnia at 2 weeks (Supplementary Table S3). There was no significant difference in changes in FSS scores from baseline between the 2 intervention groups [−1.62 (1.29) vs. -1.07 (1.21), *p* = 0.756 for 1 week, −4.24 (1.22) vs. -1.53 (1.11), *p* = 0.105 for 2 weeks, Figure 3F]. Subgroup analysis revealed that the effect of 2-week K56 intervention on FSS scores was not statistically significant across any subgroup (males, females, young adults, and older adults), which is consistent with the findings in the general population. For ISI scores, the K56 intervention demonstrated more pronounced effects in females [−2.00 (0.56) vs. 0.21 (0.53), *p* = 0.005, Supplementary Table S4] and younger participants [−1.68 (0.59) vs. 0.90 (0.67), *p* = 0.005, Supplementary Table S5], whereas no significant effects were observed in males and older adults (Supplementary Tables S4, S5).

**Figure 4:**
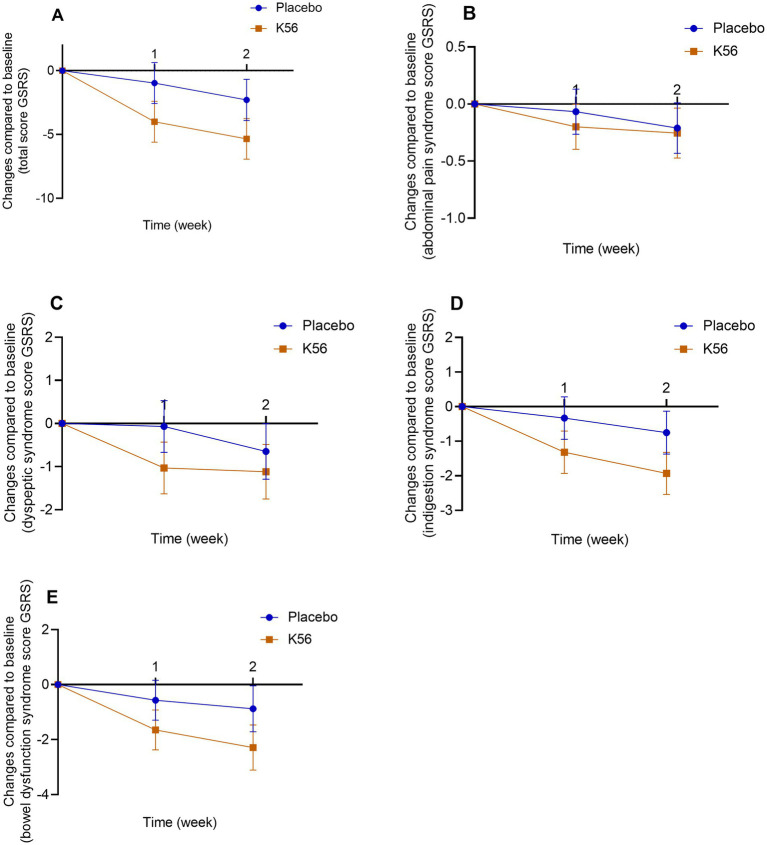
Changes and standard errors in self-report gastrointestinal symptom scores between the K56 group and the placebo group from baseline over 2 weeks. **(A)** total GSRS score, **(B)** GSRS abdominal pain syndrome, **(C)** GSRS dyspeptic syndrome, **(D)** GSRS indigestion syndrome, **(E)** GSRS bowel dysfunction. If a statistically significant difference in the changes of symptom score at week 1 or 2 from baseline between the two groups is observed, it will be indicated by an asterisk. In the absence of an asterisk, it signifies that no significant difference has been detected between the groups. PSS, Cohen’s perceived stress scale; DASS, the depression, anxiety and stress scales; GSRS, the gastrointestinal symptom rating scale; ISI, the insomnia severity index; FSS, the fatigue severity scale.

Results for self-report gastrointestinal symptoms between the K56 group and the placebo group are presented in Figure 4. There were no significant difference in the changes of total GSRS score [−5.34 (1.19) vs. -2.28 (1.07), *p* = 0.059, Figure 4A], GSRS abdominal pain syndrome score [−0.25 (0.16) vs. -0.21 (0.15), *p* = 0.843, Figure 4B], GSRS dyspeptic syndrome score [−1.12 (0.53) vs. -0.65 (0.36), *p* = 0.461, Figure 4C], GSRS indigestion syndrome score [−1.93 (0.47) vs. -0.75 (0.39), *p* = 0.058, Figure 4D], or GSRS bowel dysfunction score [−2.29 (0.54) vs. -0.88 (0.62), *p* = 0.091, Figure 4E] from baseline to 2 weeks between the two intervention groups. Paired within-group *post hoc* analysis revealed a statistically significant decrease in three GSRS domain scores in the K56 group [10.83 (0.41) vs. 13.03 (0.51), *p* < 0.001 for bowel dysfunction syndrome, 9.19 (0.46) vs. 11.07 (0.57), *p* < 0.001 for indigestion syndrome, and 8.02 (0.43) vs. 9.01 (0.48), *p* = 0.006 for dyspeptic syndrome, Supplementary Table S3], which was not observed in placebo group. In the subgroup analysis stratified by age, no significant differences between subgroups were observed in the effect of K56 intervention on the total GSRS score or the scores across four syndrome dimensions. In the subgroup analysis by sex, the K56 (vs placebo) intervention appeared to have a more pronounced effect in females, particularly evident in the total GSRS scores [−5.96 (1.58) vs. -1.35 (1.20), *p* = 0.018] and GSRS bowel dysfunction scores [−2.52 (0.65) vs. -0.21 (0.71), *p* = 0.018, Supplementary Table S4].

### 3.4 Adverse events

No clinically significant adverse events were reported throughout the entire study.

### 3.5 Serum biomarkers

**Figure 5:**
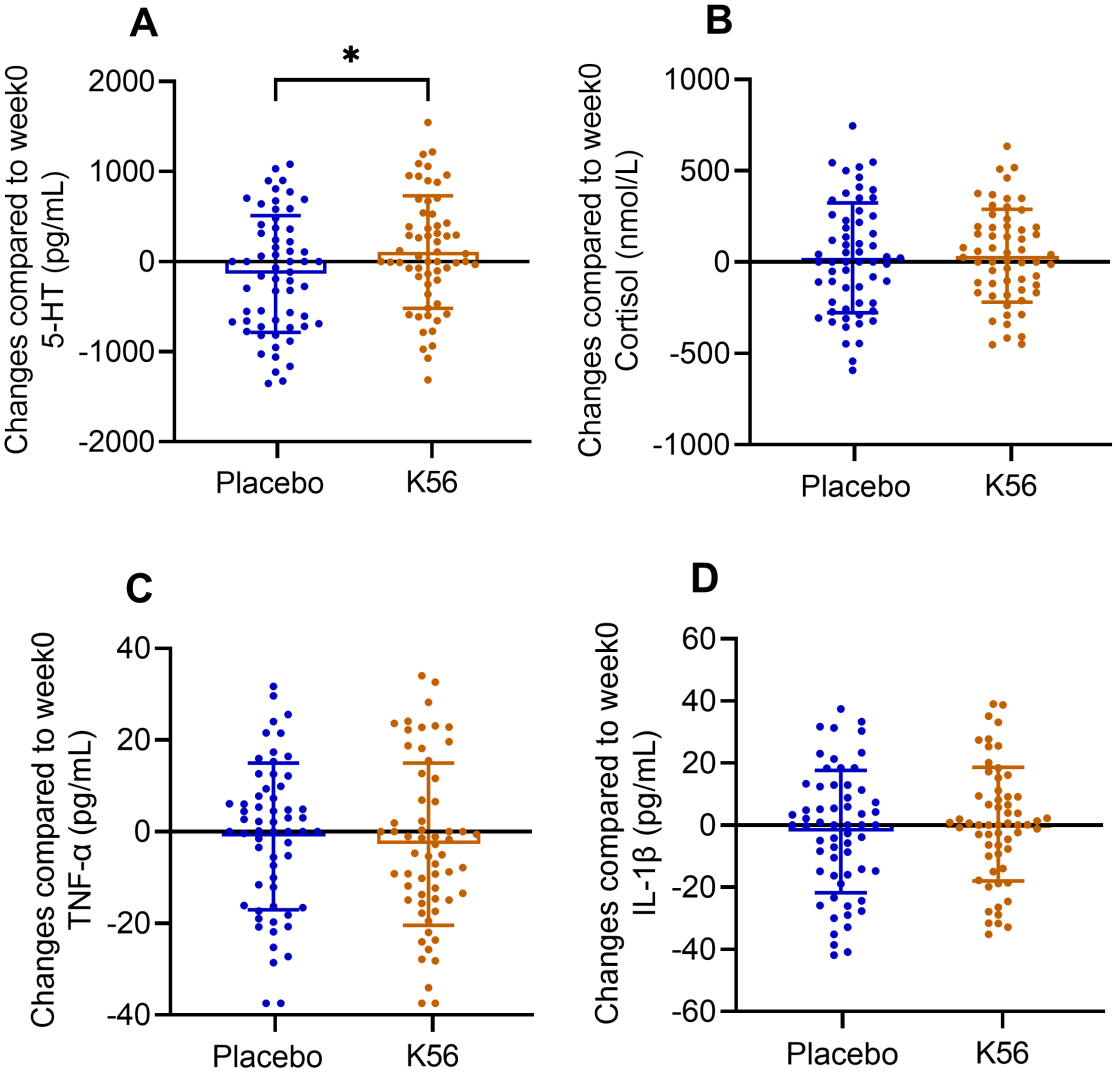
Changes and standard errors in serum neurotransmission and inflammation markers between the K56 group and the placebo group from baseline to 2 weeks. **(A)** 5-hydroxytryptamine (5-HT), **(B)** cortisol, **(C)** TNF-*α*, and **(D)** IL-1β. The asterisk denotes the statistical significance of the differences between groups in changes observed at 2 weeks compared to the baseline. ^*^p < 0.05.

The results of serum markers, including 5-HT, cortisol, TNF-*α*, and IL-1β, are presented in Figure 5. Following a two-week intervention period, the K56 group exhibited a significantly greater increase in 5-HT compared to the placebo group [115.06 (86.31) vs. -148.28 (91.22), *p* = 0.038, Figure 5A]. However, no significant between-group differences were observed for the other three measures in terms of their change values after the intervention [36.35 (34.85) vs. 24.65 (42.39), *p* = 0.831 for cortisol, −2.91 (2.45) vs. -1.12 (2.25), *p* = 0.591 for TNF-*α*, 0.39 (2.53) vs. -2.26 (2.78), *p* = 0.483 for IL-1β, Figures 5B–D].

### 3.6 Gut microbiome analysis

**Figure 6:**
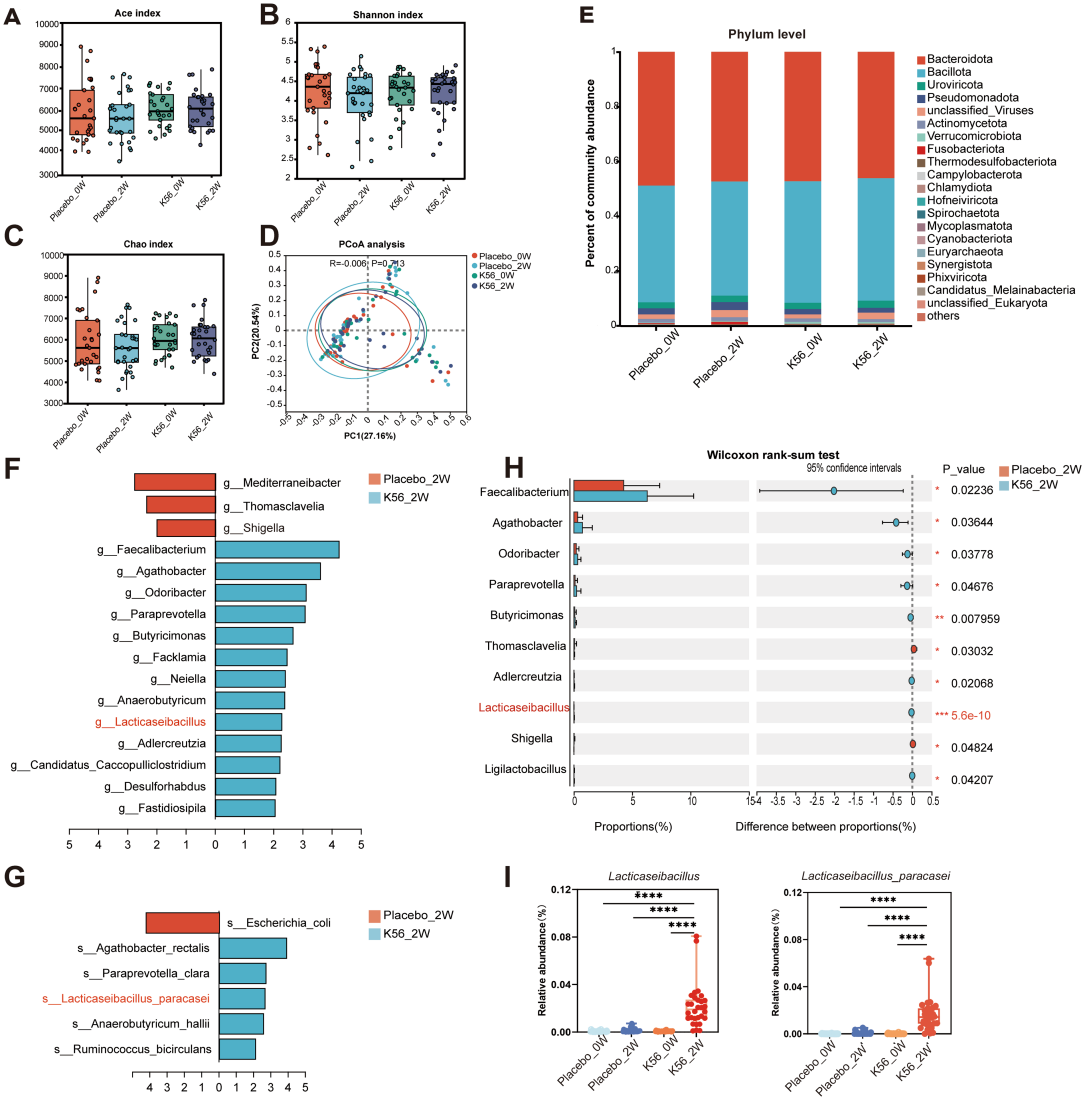
Effects of K56 intervention on the composition characteristics of gut microbiota in fecal samples. **(A)** Alpha diversity assessed by Ace index; **(B)** Alpha diversity assessed by Shannon index; **(C)** Alpha diversity assessed by Chao index; **(D)** Principal coordinates analysis (PCoA) based on un-weighted UniFrac distances of gut microbiota composition; **(E)** Relative abundances of main phyla; **(F)** Significantly different microbiota genera between groups identified by LEfSe analysis (*p* < 0.05, LDA score > 2); **(G)**Significantly different microbiota species between groups identified by LEfSe analysis (*p* < 0.05, LDA score > 2); **(H)** Significantly different microbiota genera between placebo and K56 group after intervention; **(I)** Boxplots of *Lacticaseibacillus* and *Lacticaseibacillus parasei*. The significance levels of the comparisons between the changes in one group relative to the other three groups were calculated using the two-side Wilcoxon rank-sum test and are denoted as follows: *p < 0.05; **p < 0.01,***p < 0.001. Placebo_0W: samples of the placebo group at baseline, Placebo_2W: samples of the placebo group after 2 weeks intervention, K56_0W: samples of the probiotic group at baseline, K56_2W: samples of the K56 group after 2 weeks intervention.

To assess the impact of K56 treatment on gut microbiota, metagenomic sequencing was performed on 120 stool samples, resulting in an average of 72,927,438 reads per sample. The composition characteristics of gut microbiota are shown in Figure 6. Our results revealed no significant changes within group or differences between groups in terms of Alpha-diversity (*p* > 0.05, Figures 6A–C) and Beta-diversity (*p* > 0.05, Figure 6D) following the intervention. The most abundant phyla were *Bacillota* and *Bacteroidota*, accounting for 48 and 43% percentage of the total bacterial communities, respectively. At the phylum level, there was no substantial impact observed after intervention in the K56 group (Figure 6E). Further liner discriminat effect size (*LefSe*) analysis (*p* < 0.05, LDA > 2) confirmed that *Faecalibacterium, Agathobacter, Odoribacter, Paraprevotella, Butyricimonas* and *Anaerobutyricum* were significantly enriched bacteria genus in the K56 group, while in the placebo group, these genus were *Shigella*, *Thomasclavelia*, and *Mediterraneibacter* (Figure 6F). At the species level, the abundances of *Lacticaseibacillus paracasei*, *Anaerobutyricum hallii, Ruminococcus callidus, Paraprevotella clara*, and *Agathobacter rectails* were significantly enriched in the K56 group after intervention, while some harmful bacteria such as *Escherichia coli* was significantly enriched in the placebo group (Figure 6G). Notably, at baseline, there were no statistically significant difference in the relative abundance of *Lacticaseibacillus* at the genus level *and Lacticaseibacillus parasei* at the species level between the two groups. After the interventon, the relative abundance of *Lacticaseibacillus* (0.0217% in the K56 group vs. 0.0015% in the placebo group, representing a 14.5-fold level over the latter, *p* < 0.001) and *Lacticaseibacillus parasei* (0.0168% in the K56 group vs. 0.0007% in the placebo group, representing a 24-fold level over the latter, *p* < 0.001) increased significantly in the subjects taking K56 supplementation compared to those in the placebo group (Figures 6H,I). These results indicate that two weeks of K56 supplementation significantly modulated the gut microbiota composition.

### 3.7 Fecal metabolites analysis

**Figure 7:**
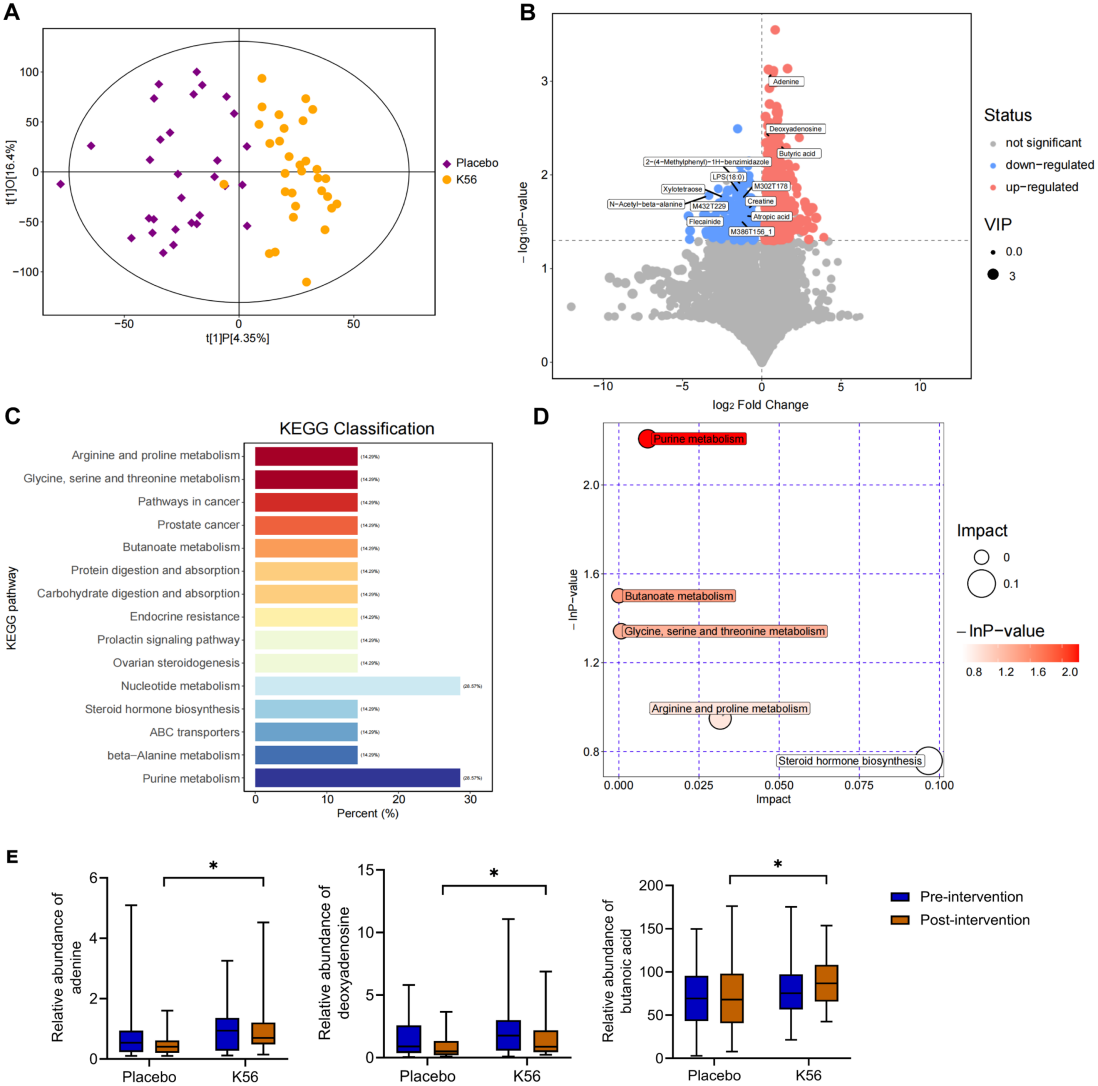
Effects of K56 intervention on the fecal metabolites. **(A)** OPLS-DA analysis in K56 and placebo groups after intervention; **(B)** Volcanic maps of differential metabolites between two groups, with the red, blue, and gray maps representing significantly upregulated, downregulated (*p* < 0.05) and unchanged metabolites, the horizontal axis representing the fold change (log^2^ fold change) of metabolites in different groups, while the vertical axis representing the significance level of the difference (−log 10 *p* value); **(C,D)** Pathway Enrichment analysis of differentially metabolites between the K56 and the placebo groups; **(E)** The concentration of ademine, deoxyadenosine, and butyric acid in the feces in the K56 and the placebo groups. OPLS-DA, orthogonal partial least squares discriminant analysis; *p < 0.05, compared with the placebo group.

The effects of the K56 intervention on fecal metabolites were further analyzed by conducting non-targeted LC–MS based metabolomics on these 120 stool samples. As shown in Figure 7A, the OPLS-DA score scatter plot demonstrates a distinct separation between the K56 and placebo groups, indicating differences in gut metabolic profiles between groups after intervention. A total of 24,910 metabolites were identified in the K56 and placebo group before and after intervention. Based on variable importance in the projection (VIP) values >1 and *p* < 0.05, 118 differentially accumulated metabolites between the two group were identified, of which 94 metabolites were enriched and 24 metabolites were depleted (Figure 7B). These metabolites were subjected to subsequent KEGG analysis, revealing that the pathways of Purines metabolism and butanoate metabolism were the most significantly affected pathways by K56 intervention (Figures 7C,D). Specifically, within the Purine metabolism pathway, the relative abundance of adenine and deoxyadenosine was significantly higher in the K56 group compared to the placebo group. In addition, a significant increase in butyric acid relative abundance was observed within the butanoate metabolism pathway, for the K56 group relative to the placebo group (Figure 7E).

**Figure 8:**
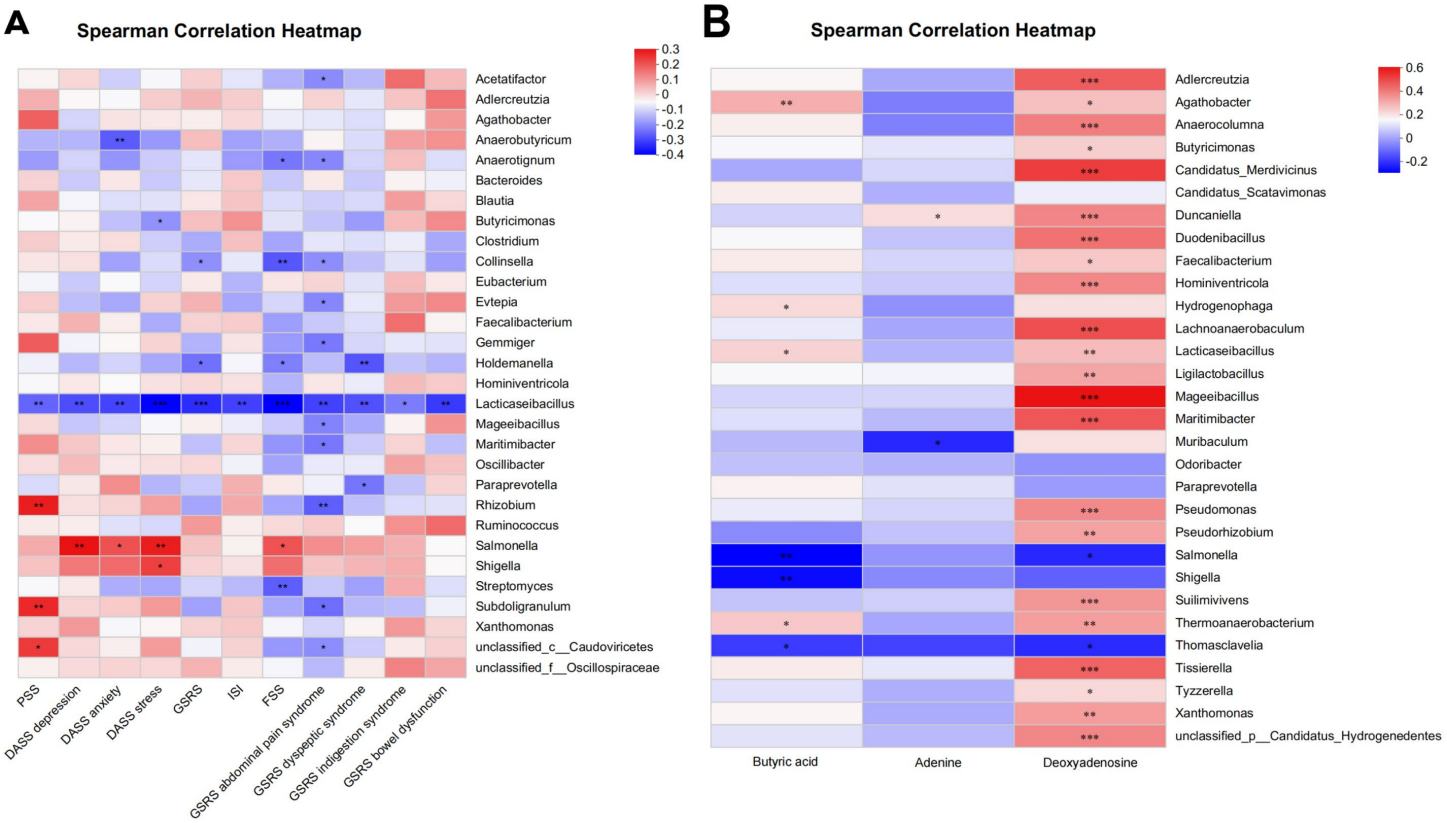
Association analysis between phenotypic characteristics, microbiota, and metabolite profiles by partial Spearman correlation. **(A)** Spearman correlation heatmap of microbiota and phenotypic characteristics. **(B)** Spearman correlation heatmap of microbiota and metabolite profiles. *p < 0.05; **p < 0.01 (non-significant data in all comparisons are omitted). PSS, Cohen’s perceived stress scale; DASS, the depression, anxiety and stress scales; GSRS, the gastrointestinal symptom rating scale; ISI, the insomnia severity index; FSS, the fatigue severity scale.

The questionnaire indicators, along with the top 30 bacteria genera exhibiting statistically significant inter-group difference, and the differentially abundant gut metabolites between groups were selected for Spearman correlation analysis. The results revealed a significant negative correlation between the increase of *Lacticaseibacillus* relative abundance and higher scores of almost all psychological and gastrointestinal symptoms (all *p* < 0.05) (Figure 8A). Moreover, among the differentially abundant metabolites of the two groups, the increase of butyric acid exhibited positive correlation with the enrichment of *Lacticaseibacillus* (*r* = 0.2, *p* = 0.03) (Figure 8B).

## 4 Discussion

The current study represents one of the few studies to investigate stress alleviation strategies in a specific population-master’s and doctoral students facing graduation pressure. The results demonstrated that compared to the placebo group, a 2-week administration of *Lacticaseibacillus paracasei* K56 potentially exerted beneficial effects in alleviating perceived stress (based on DASS stress scores), while showing significant beneficial effects in reducing anxiety and insomnia symptoms, along with a significant elevation in serum 5-HT levels. These effect may be correlated with the modulation of the abundance of beneficial bacteria (*Lacticaseibacillus*) and specific metabolites (butyric acid).

Exposure to stress can impact the gut barrier and microbial composition, and alterating of the gut microbiota by probiotics interventionis maybe a novel approach to influencing stress, mood and well-being (39). A meta-analysis has shown that probiotics can reduce subjective stress level in healthy volunteers and may alleviate stress-related sub-threshold anxiety level (40). However, the beneficial effects of probiotics are strain-specific (41), as even the same strain produced different effects depending on populations or stressful environments. Previous research has shown that consumption of *Lacticaseibacillus paracasei* Lpc-37 for 5 weeks significantly reduced perceived stress in healthy adults with any source of stress (12), but not in healthy students facing exam stress (13). Master’s and doctoral students are highly susceptible to stress; however, there is limited research on stress alleviation strategies for this population. In our study, two validated questionnaires, PSS-10 (20) and DASS-21 (23), were used to assess the levels of stress among this population.The findings demonstrated that compared to the placebo group, the 2-week K56 intervention resulted in a significant reduction in DASS stress scores but had no significant effect on PSS scores, which is consistent with previous studies of a similar nature (13, 42, 43), and the discrepancy between the two questionnaire outcomes may be attributed to differences in the assessment properties of the two instruments. Although the PSS score, as the primary outcome, did not show significant differences, two studies targeting stressed student populations reported similar results to ours (13, 44). The variation in PSS scores compared to Patterson et al.’s study (12) may be attributed to differences in probiotic strains, dosages, treatment duration, and study populations. These results suggest that K56 exhibits substantial potential for alleviating stress within this particular population.

The presence of stress can contribute to the development of depression, anxiety, and other related conditions. In this study, a 2-week K56 intervention demonstrated significant improvements in anxiety symptoms but had no substantial impact on depression, consistent with previous studies showing that probiotics alleviate anxiety but not depression (42, 43). Stress has also been found to be a major contributing factor of the insomnia. This study found a significant improvement in insomnia symptoms measured by the ISI scale following treatment with K56. To our knowledge, this is one of the few studies reporting a reduction in insomnia scores using ISI; however, further extensive clinical trials are warranted to validate the potential advantages of probiotics on sleep. In addition, psychological stress is directly related to the onset of gastrointestinal symptoms (45). Although improvements in gastrointestinal symptoms were observed after intervention compared to before intervention, there was no significant difference in GSRS scores between the K56 and placebo groups. We hypothesize that this lack of significance may be attributed to baseline levels of gastrointestinal health prior to the study initiation or the relatively short duration of probiotic intervention.

Previous studies have shown that 5-HT is closely associated with stress symptoms (46). More than 90% of 5-HT is synthesized in enterocytes, with 2% entering the bloodstream to affect stress perception by acting on serotonin receptors in neurons of brain (47, 48). In our study, participants who consumed K56 fermented milk beverage exhibited higher 5-HT change values compared with the placebo group. These results suggest that 5-HT may play a key role in regulating stress response mediated by K56. While, cortisol, another stress-related biomarker (49, 50), did not showed significant changes following K56 intervention, which is consistent with other studies (42, 43). Considering that cortisol release is a complex process affected by multiple factors, such as diet and exercise habits may also contribute to variations in cortisol release. The presence of stress often triggers inflammatory response (51), and the modulation of the inflammatory pathway is also a key mechanisms through which probiotics enhance health (52). A previous study has demonstrated that K56 reduces serum levels of IL-1β and TNF-*α* in mice (16). However, in our study, contrary to expectations, 2 weeks of K56 intervention did not yield significant changes in serum IL-1β and TNF-α levels. It is highly likely that the intervention period in this study was not long enough for observing measurable effects on serum markers.

Recent studies have shown that depletion of gut microbiota especially *Lactobacillus* disrupts the rhythmicity of stress pathways in the brain (39, 53–55), suggesting that targeting *Lactobacillus* in gut microbiota could be a potential strategy for alleviating stress. In this study, supplementation with K56 significantly increased the abundance of *Lacticaseibacillus* at both genus and species levels. Correlation analysis revealed a significant negative association between *Lacticaseibacillus* and almost all symptom scores, strongly supporting its role in relieving stress and related symptoms. In addition, K56 intervention also resulted in increases in the relative abundance of several strains including *Odoribacter* which have been found to be abundant during aging and may have potential anti-inflammatory properties and support brain health (53, 56), as well as *Paraprevotella* whose reduced abundance has been observed in individuals with depression (57, 58). Furthermore, K56 consumption reduced *Shigella* at the genus level and *E.coli* at the species level, which have been linked to the “leaky gut,” triggering systemic inflammation and may contribute to stress-related symptoms (59). Overall, our results highlighted the beneficial impacts of K56 supplementation on the gut microbiota.

The composition of the gut microbiota determines the levels of intestinal metabolites. In our study, we observed that K56 intervention significantly impacted purine metabolism and butanoate metabolism, with notable increases in adenine and deoxyadenosine within the former pathway, and elevated levels of butyric acid within the latter pathway. Previous studies have demonstrated a prominent association between adenine levels and chronic stress-induced depression in mouse models (60), and suggests a role in the regulation of sleep–wake cycle (61). Butyrate serves as a crucial energy source for colonocytes and plays a vital role in maintaining the intestinal barrier and blood–brain integrity (62). Reigstad et al. found that butyrate could also promote the transcription of tryptophan hydroxylase 1 (Tph1) in enterochromaffin cells, improving the production of 5-HT (63). Therefore, the alleviation of stress, anxiety, and insomnia symptoms observed in this study may also be attributed to the increased 5-HT levels mediated by butyric acid through this pathway. Spearman’s correlaiton analysis with differential gut microbiota revealed that only butyric acid exhibited a strong positive correlation with the abundance of *Lacticaseibacillus,* indicating its potential significance as a key metabolite associated with K56 intervention in this study.

To the best of our knowledge, this study represents one of the few studies in addressing the unique stress experienced by master’s and doctoral students during their graduation period, and the K56 shows potential benefits for this group. However, there are several limitations. Firstly, participants were recruited from a limited geographic area (Beijing, China), which may restrict the generalizability of our findings to broader populations. Secondly, there was a significantly higher proportion of female participants in this study, and an imbalance in the gender ratio could potentially affect the results. Thirdly, although we restricted antibiotic and probiotic/prebiotic usage, strict dietary controls were not imposed on the participants. Fourthly, since no previous clinical trials have investigated the stress-alleviating effects of K56, the sample size for this study was estimated based on results from similar clinical studies of other *Lacticaseibacillus paracasei* strains, which may have led to an imprecise estimation of the required sample size. Fifth, we only measured and analyzed the changes of two serum inflammatory factors, TNF-*α* and IL-1β, it would be valuable to consider measuring multiple inflammatory factors. Furthermore, the confirmation of whether the potential butyric acid identified in this study serves as a pivotal metabolite for the stress relief effect of K56 intervention necessitates further investigations. Finally, the post-subgroup analysis of this study indicates potential heterogeneity in the intervention effect of K56 across different genders and age groups. However, as this study was not powered to detect differences within subgroups, these exploratory findings should be interpreted with caution. Future research should include more comprehensive clinical trials specifically designed to investigate and confirm these preliminary observations.

## 5 Conclusion

Our study demonstrates that a 2-week intervention with *Lacticaseibacillus paracasei* K56 may potentially alleviate stress and significantly ameliorate associated symptoms, including anxiety and insomnia, in master’s and doctoral students experiencing graduation-related stress. These beneficial effects could potentially be attributed to the upregulation of beneficial gut microbiota and increased levels of the key metabolite butyric acid. Furthermore, K56 treatment exhibited excellent tolerability and safety profile with minimal occurrence of adverse events. These findings offer valuable insights into the potential application of short-term psychobiological interventions for stress management in specific populations. However, to comprehensively elucidate its therapeutic efficacy and underlying mechanisms, future investigations should encompass large-scale, long-term randomized controlled trials.

## Data Availability

The raw data supporting the conclusions of this article will be made available by the authors, without undue reservation.
