# Supplementary material for: Effects of Lacticaseibacillus paracasei K56 on perceived stress among pregraduate students: a double-blind, randomized, placebo-controlled trial
Source: Front Nutr. 2025 Mar 12;12:1544713. doi: 10.3389/fnut.2025.1544713 (PMC11936786; doi:10.3389/fnut.2025.1544713)
Supplement: Supplementary file 1 [file Table_1.DOCX]

Supplementary Material

# Supplementary Data

## Supplementary methods for Fecal sample metagenome and metabolome detection

### Metagenomic analysis

Fecal samples were selected from 60 of the 120 participants who completed the entire trial and were used for single-end metagenomics sequencing based on the BGISEQ-500 platform at BGI Genomics (Shenzhen, China). DNA libraries were prepared by using 500 ng of input DNA. DNA was ultrasonically fragmented by using a E220 Focused-ultrasonicator (Covaris, UK), yielding 300–700-bp fragments. Products were purified with an AxyPrep Mag PCR Clean-Up Kit (Axygen Scientific, Inc., Union City, CA, USA) and eluted with 45 μl TE buffer. Afterward, 20 ng of purified DNA was processed with end-repairing and A-tailing by using a 2:2:1 mixture of T4 DNA polymerase (ENZYMATICS™ P708-1500), T4 polynucleotide kinase (ENZYMATICS™ Y904-1500), and rTaq DNA polymerase (TAKARA™ R500Z). Adaptors with specific barcodes were ligated to the DNA fragment by T4 DNA ligase (ENZYMATICS™ L603-HC-1500) at 23°C, followed by PCR amplification. Finally, a single-strand circular DNA library was generated using 55 ng of purified PCR products by denaturing at 95°C and circular ligation using T4 DNA ligase (ENZYMATICS™ L603-HC-1500) at 37°C. Equal amounts of 8 barcoded libraries were pooled for the generation of DNA Nanoballs (DNB) and loaded onto one lane for sequencing using the BGISEQ-500 platform.Sequencing was performed according to the BGISEQ-500 protocol (No. CNGB-DP-SOP10-002) employing the SE50 mode with a following base calling process to remove adaptors automatically (1). Discard low-quality reads, and the bacterial reads aligned to the integrated gene catalogue (IGC) 9.9M reference to obtain a normalized gene abundance profile, as described previously (2). The metagenomic species (MGS) profile was generated based on a previously described procedure (3). Annotation of MGSs was updated with extra information by a random forest-based method and curated manually.

### Non-target metabolic

Fecal metabolites extration and analysis followed a published method with modification(4). Briefly, metabolic extracts were obtained from fecal samples following methanol-assisted protein precipitation and then analyzed by an UHPLC system (Vanquish, Thermo Fisher Scientific) with a UPLC BEH Amide column (2.1 mm × 100 mm, 1.7 μm) coupled to the Q Exactive HFX mass spectrometer (Orbitrap MS, Thermo) (5). Mobile Phase A was H2O containing 25 mmol/L ammonium acetate and 25 mmol/L ammonia hydroxide (pH = 9.75), and Mobile Phase B was acetonitrile. The auto-sampler temperature was set at 4 °C, and the injection volume was 2 μL. The sample sequence was random. The MS/MS spectra were acquired by the acquisition software Xcalibur (Thermo Fisher Scientific) on information-dependent acquisition (IDA) mode. The ESI source conditions were configured as follows: sheath gas flow rate at 30 Arb, Aux gas flow rate at 25 Arb, capillary temperature at 350 °C, full MS resolution at 120,000, MS/MS resolution at 7,500, collision energy at 10/30/60 in NCE mode, and spray voltage at 3.6 kV (positive) or −3.2 kV (negative). The raw data were converted to the mzXML format using ProteoWizard and processed with R package xcms (6), for peak detection, extraction, alignment, and integration. The in-house MS2 database was applied for metabolite identification. The OPLS-DA was performed using the statistics function prcomp in R (version 4.0.2). In order to avoid overfitting, a permutation test (200 permutations) was performed. The discriminative metabolites between the two groups were identified with variable importance in the projection (VIP) > 1 (determined by OPLS-DA) and P < 0.05 (determined by Student’s t test). The KEGG database ([http://www.genome.jp/kegg/](http://www.genome.jp/kegg/)) and MetaboAnalyst database ([http://www.metaboanalyst.ca/](http://www.metaboanalyst.ca/)) were used for pathway enrichment analysis.

## Ethics

No further review was required since, given the nature of the intervention, no harms or side effects were recorded and no amendments in the study protocol were made.

# Supplementary Figures and Tables

**Table S1** Study’s inclusion and exclusion criteria.

| **Inclusion criteria** |
| --- |
| 1) Age: 18-35 years old. |
| 2) Master's or doctoral students who were expected to graduate between June 2024 and July 2024. |
| 3) Measured as moderate stress by Cohen's Perceived Stress Scale (PSS-10). |
| 4) Signed informed consent form for participation. |
| **Exclusion criteria** |
| 1) Individuals with other stress sources apart from graduation, such as family, illness, finance, etc. |
| 2) Patients with mental disorders (e.g. depression, anxiety disorder, bipolar spectrum disorder, or schizophrenia). |
| 3) Patients with severe gastrointestinal diseases (e.g. gastric ulcers, Crohn's disease, or ulcerative colitis) or chronic illness (e.g. diabetes, liver disease, kidney disease, or heart disease). |
| 4) On psychoactive medication, gastrointestinal medication, dietary supplements, or probiotics; |
| 5) Individuals with a recent history of antibiotic treatment. |
| 6) Those engaging in excessive smoking, alcohol consumption, or drug abuse. |
| 7) Pregnant or lactating women, or those allergic to the test sample. |
| 8) Already undergoing other stress-relieving therapies (e.g. cognitive behavioral therapy, music therapy, etc.). |
| 9) Persons with irregular schedules that could hinder the implementation of intervention strategies. |
| 10) Participants who have been involved in other clinical trials within the last 30 days or are currently enrolled in one. |

**Table S2** Composition of test beverages

|  | Placebo milk | K56 fermented milk |
| --- | --- | --- |
| Energy (kJ/100mL) | 284 | 284 |
| Protein (g/100 mL) | 1.1 | 1.1 |
| Fat (g/100 mL) | 0 | 0 |
| Carbohydrate (g/100 mL) | 15.6 | 15.6 |
| Na (mg/100 mL) | 30 | 30 |
| *Lacticaseibacillus paracasei* K56 (CFU/100 mL) | 0 | 6.0 × 10^10^ |

**Table S3** Alternations in self-report psychological and gastrointestinal symptoms after probiotic supplementation

| Parameters | Group | 0 w | 1 w | *2 w* |
| --- | --- | --- | --- | --- |
| PSS | K56 | 20.13 ± 4.36 | 18.70 ± 4.26 *** | 18.46 ± 4.15 ** |
|  | Placebo | 19.90 ± 4.30 | 19.58 ± 4.32 | 19.74 ± 4.27 |
| DASS depression | K56 | 6.00 (3.00, 9.00) | 5.00 (3.00, 7.00) * | 4.00 (1.00, 7.00) *** |
|  | Placebo | 5.50 (3.00, 8.00) | 5.00 (3.00, 7.00) | 4.00 (2.00, 6.00) ** |
| DASS anxiety | K56 | 6.00 (3.00, 9.00) | 4.00 (3.00, 7.00) | 3.00 (2.00, 6.00) *** |
|  | Placebo | 5.00 (3.00, 7.00) | 5.00 (3.00, 7.00) | 4.00 (3.00, 6.00) |
| DASS stress | K56 | 8.00 (6.00, 10.00) | 7.00 (5.00, 9.00) ** | 6.00 (3.00, 8.00) *** |
|  | Placebo | 8.00 (6.00, 10.00) | 7.50 (5.00, 9.00) | 6.00 (5.00, 8.50) *** |
| ISI | K56 | 8.25 ± 4.70 | 6.80 ± 4.09 ** | 6.51 ± 4.01 *** |
|  | Placebo | 7.22 ± 4.34 | 7.13 ± 4.48 | 7.25 ± 4.10 |
| FSS | K56 | 43.48 ± 10.39 | 41.87 ± 11.28 | 39.36 ± 10.98 ** |
|  | Placebo | 44.45 ± 9.96 | 43.38 ± 11.90 | 43.60 ± 10.59 |
| GSRS | K56 | 33.17 ± 9.83 | 29.17 ± 7.85 *** | 28.03 ± 7.85 *** |
|  | Placebo | 33.6 ± 10.29 | 32.63 ± 11.35 | 31.28 ± 10.37 * |
| GSRS abdominal pain syndrome | K56 | 1.00 (1.00, 2.00) | 1.00 (1.00, 2.00) | 1.00 (1.00, 2.00) |
|  | Placebo | 2.00 (1.00, 3.00) | 1.50 (1.00, 2.00) | 2.00 (1.00, 2.00) |
| GSRS dyspeptic syndrome | K56 | 8.00 (6.00, 11.00) | 8.00 (6.00, 10.00) ** | 7.00 (6.00, 9.00) ** |
|  | Placebo | 9.00 (7.00, 11.00) | 8.50 (6.25, 10.00) | 8.00 (6.00, 10.00) |
| GSRS indigestion syndrome | K56 | 10.00 (8.00, 13.00) | 9.00 (7.00, 11.00) ** | 9.00 (7.00, 11.00) *** |
|  | Placebo | 11.00 (8.00, 13.75) | 10.00 (7.00, 13.00) | 10.00 (7.00, 12.00) |
| GSRS bowel dysfunction | K56 | 13.00 (9.25, 16.00) | 11.00 (9.25, 14.00) ** | 10.00 (8.00, 13.00) *** |
|  | Placebo | 13.00 (9.00, 17.00) | 12.00 (9.00, 16.00) | 11.00 (8.50, 17.00) |

Values represent mean±standard deviation and median (25th, 75th percentiles). * denotes the after-intervention difference (1 w or 2 w compared with baseline,* *P*<0.05,** *P*<0.01, ****P*<0.001. w, week; PSS, Cohen's perceived stress scale; DASS, the depression, anxiety and stress scales; GSRS, the gastrointestinal symptom rating scale; ISI, the insomnia severity index; FSS, the fatigue severity scale.

**Table S4** Subgroup analysis by sex of effects of K56 on self-report psychological and gastrointestinal symptoms

| Parameter/Group | Change from baseline to 2 w | *P* |
| --- | --- | --- |
| PSS |  |  |
| K56-M | -1.47 ± 3.98 | 0.785 |
| Placebo-M | -1.86 ± 3.63 |  |
| K56-F | -1.75 ± 3.67 | 0.016 * |
| Placebo-F | 0.09 ± 3.28 |  |
| DASS depression |  |  |
| K56-M | -1.87 ± 3.48 | 0.588 |
| Placebo-M | -1.07 ± 4.30 |  |
| K56-F | -1.39 ± 2.68 | 0.708 |
| Placebo-F | -1.16 ± 2.86 |  |
| DASS anxiety |  |  |
| K56-M | -1.07 ± 2.25 | 0.648 |
| Placebo-M | -0.43 ± 4.82 |  |
| K56-F | -1.70 ± 2.53 | 0.001 * |
| Placebo-F | 0.21 ± 2.55 |  |
| DASS stress |  |  |
| K56-M | -1.93 ± 3.79 | 0.998 |
| Placebo-M | -1.93 ± 5.43 |  |
| K56-F | -2.23 ± 2.58 | 0.009 * |
| Placebo-F | -0.65 ± 2.90 |  |
| ISI |  |  |
| K56-M | -1.07 ± 3.59 | 0.768 |
| Placebo-M | -0.64 ± 4.05 |  |
| K56-F | -2.00 ± 3.69 | 0.005 * |
| Placebo-F | 0.21 ± 3.45 |  |
| FSS |  |  |
| K56-M | -3.07 ± 7.30 | 0.76 |
| Placebo-M | -2.07 ± 9.98 |  |
| K56-F | -4.64 ± 10.06 | 0.095 |
| Placebo-F | -1.35 ± 7.96 |  |
| GSRS |  |  |
| K56-M | -3.53 ± 6.66 | 0.571 |
| Placebo-M | -5.14 ± 8.40 |  |
| K56-F | -5.95 ± 9.82 | 0.018 * |
| Placebo-F | -1.35 ± 7.86 |  |
| GSRS abdominal pain syndrome |  |  |
| K56-M | -0.07 ± 0.80 | 0.642 |
| Placebo-M | -0.21 ± 0.89 |  |
| K56-F | -0.32 ± 1.38 | 0.694 |
| Placebo-F | -0.21 ± 1.19 |  |
| GSRS dyspeptic syndrome |  |  |
| K56-M | -0.47 ± 2.90 | 0.554 |
| Placebo-M | -1.07 ± 2.50 |  |
| K56-F | -1.34 ± 4.36 | 0.294 |
| Placebo-F | -0.51 ± 2.76 |  |
| GSRS indigestion syndrome |  |  |
| K56-M | -1.47 ± 2.64 | 0.757 |
| Placebo-M | -1.14 ± 2.93 |  |
| K56-F | -2.09 ± 3.91 | 0.054 |
| Placebo-F | -0.63 ± 3.00 |  |
| GSRS bowel dysfunction |  |  |
| K56-M | -1.60 ± 3.91 | 0.404 |
| Placebo-M | -2.93 ± 4.53 |  |
| K56-F | -2.52 ± 4.29 | 0.018 * |
| Placebo-F | -0.21 ± 4.63 |  |

Values are expressed as mean ± standard deviation, representing the changes from baseline following a two-week intervention period. *P* denotes the inter-group difference (2 w, * P<0.05,** P<0.01, ***P<0.001. w, week; PSS, Cohen's perceived stress scale; DASS, the depression, anxiety and stress scales; GSRS, the gastrointestinal symptom rating scale; ISI, the insomnia severity index; FSS, the fatigue severity scale; Placebo-M (n = 15), the male subgroup of the placebo group; K56-M (n = 16), the male subgroup of the K56 group; Placebo-F (n = 45), the female subgroup of the placebo group; K56-F (n = 44), the female subgroup of the K56 group.

**Table S5** Subgroup analysis by age of effects of K56 on self-report psychological and gastrointestinal symptoms

| Parameter/Group | Change from baseline to 2 w | *P* |
| --- | --- | --- |
| PSS |  |  |
| K56-Y | -1.76 ± 3.98 | 0.057 |
| Placebo-Y | 0.03 ± 3.54 |  |
| K56-O | -1.55 ± 3.31 | 0.494 |
| Placebo-O | -0.88 ± 3.31 |  |
| DASS depression |  |  |
| K56-Y | -1.70 ± 3.00 | 0.123 |
| Placebo-Y | -0.61 ± 2.70 |  |
| K56-O | -1.18 ± 2.70 | 0.542 |
| Placebo-O | -1.77 ± 3.72 |  |
| DASS anxiety |  |  |
| K56-Y | -1.78 ± 2.23 | 0.001 * |
| Placebo-Y | 0.32 ± 2.89 |  |
| K56-O | -1.14 ± 2.82 | 0.364 |
| Placebo-O | -0.27 ± 3.60 |  |
| DASS stress |  |  |
| K56-Y | -2.49 ± 2.86 | 0.009 * |
| Placebo-Y | -0.52 ± 3.15 |  |
| K56-O | -1.59 ± 2.94 | 0.932 |
| Placebo-O | -1.50 ± 4.21 |  |
| ISI |  |  |
| K56-Y | -1.68 ± 3.59 | 0.005 * |
| Placebo-Y | 0.90 ± 3.72 |  |
| K56-O | -1.91 ± 3.85 | 0.416 |
| Placebo-O | -1.08 ± 3.17 |  |
| FSS |  |  |
| K56-Y | -4.30 ± 9.51 | 0.081 |
| Placebo-Y | -0.42 ± 8.36 |  |
| K56-O | -4.14 ± 9.43 | 0.619 |
| Placebo-O | -2.85 ± 8.44 |  |
| GSRS |  |  |
| K56-Y | -0.65 ± 7.94 | 0.068 |
| Placebo-Y | -2.48 ± 7.88 |  |
| K56-O | -4.14 ± 10.94 | 0.458 |
| Placebo-O | -2.04 ± 8.48 |  |
| GSRS abdominal pain syndrome |  |  |
| K56-Y | -0.22 ± 1.20 | 0.574 |
| Placebo-Y | -0.06 ± 0.96 |  |
| K56-O | -0.32 ± 1.36 | 0.862 |
| Placebo-O | -0.38 ± 1.27 |  |
| GSRS dyspeptic syndrome |  |  |
| K56-Y | -1.59 ± 3.48 | 0.178 |
| Placebo-Y | -0.55 ± 2.72 |  |
| K56-O | -0.32 ± 4.80 | 0.685 |
| Placebo-O | -0.77 ± 2.70 |  |
| GSRS indigestion syndrome |  |  |
| K56-Y | -2.35 ± 3.30 | 0.063 |
| Placebo-Y | -0.94 ± 2.78 |  |
| K56-O | -1.23 ± 4.08 | 0.516 |
| Placebo-O | -0.54 ± 3.22 |  |
| GSRS bowel dysfunction |  |  |
| K56-Y | -2.11 ± 3.98 | 0.284 |
| Placebo-Y | -1.00 ± 4.48 |  |
| K56-O | -2.59 ± 4.58 | 0.192 |
| Placebo-O | -0.73 ± 5.06 |  |

Values are expressed as mean ± standard deviation, representing the changes from baseline following a two-week intervention period. *P* denotes the inter-group difference (2 w, * P<0.05,** P<0.01, ***P<0.001. w, week; PSS, Cohen's perceived stress scale; DASS, the depression, anxiety and stress scales; GSRS, the gastrointestinal symptom rating scale; ISI, the insomnia severity index; FSS, the fatigue severity scale; Placebo-Y (n = 33), the younger age subgroup (age ≤ 24) of the placebo group; K56-Y (n = 37), the younger age subgroup (age ≤ 24) of the K56 group; Placebo-O (n = 27), the older age subgroup (age > 24) of the placebo group; K56-O (n = 23), the older age (age > 24) subgroup of the K56 group.

**Supplementary reference**

1. Huang J, Liang X, Xuan Y, Geng C, Li Y, Lu H, et al. A Reference Human Genome Dataset of the Bgiseq-500 Sequencer. *GigaScience* (2017) 6(5):1-9. Epub 2017/04/06. doi: 10.1093/gigascience/gix024.

2. Li J, Jia H, Cai X, Zhong H, Feng Q, Sunagawa S, et al. An Integrated Catalog of Reference Genes in the Human Gut Microbiome. *Nature biotechnology* (2014) 32(8):834-41. Epub 2014/07/07. doi: 10.1038/nbt.2942.

3. Nielsen HB, Almeida M, Juncker AS, Rasmussen S, Li J, Sunagawa S, et al. Identification and Assembly of Genomes and Genetic Elements in Complex Metagenomic Samples without Using Reference Genomes. *Nature biotechnology* (2014) 32(8):822-8. Epub 2014/07/07. doi: 10.1038/nbt.2939.

4. He J, Jin Y, He C, Li Z, Yu W, Zhou J, et al. Danggui Shaoyao San: Comprehensive Modulation of the Microbiota-Gut-Brain Axis for Attenuating Alzheimer's Disease-Related Pathology. *Frontiers in pharmacology* (2023) 14:1338804. Epub 2024/01/29. doi: 10.3389/fphar.2023.1338804.

5. Wang J, Zhang T, Shen X, Liu J, Zhao D, Sun Y, et al. Serum Metabolomics for Early Diagnosis of Esophageal Squamous Cell Carcinoma by Uhplc-Qtof/Ms. *Metabolomics* (2016) 12(7). doi: 10.1007/s11306-016-1050-5.

6. Smith CA, Want EJ, O'Maille G, Abagyan R, Siuzdak G. Xcms: Processing Mass Spectrometry Data for Metabolite Profiling Using Nonlinear Peak Alignment, Matching, and Identification. *Analytical chemistry* (2006) 78(3):779-87. Epub 2006/02/02. doi: 10.1021/ac051437y.
